# A novel 3D technique to assess symmetry of hemi pelvises

**DOI:** 10.1038/s41598-020-75884-y

**Published:** 2020-11-02

**Authors:** Peyman Bakhshayesh, Ahmed Zaghloul, Benjamin Michael Sephton, Anders Enocson

**Affiliations:** 1 Department of Molecular Medicine and Surgery, Karolinska Institute, Stockholm, Sweden; 2 Imperial College Healthcare, London, UK; 3 Barts Healthcare, London, UK

**Keywords:** Anatomy, Health care, Medical research

## Abstract

Anatomical reconstruction of pelvic fractures has been shown to affect functional outcome. Using the contra lateral side of the extremities to create a template for an ipsilateral reconstruction is common practice in orthopedic surgery. We aimed to assess whether hemi pelvises are symmetrical in terms of translation and rotation using 3D reconstruction, point to point mirroring and merging of the 3D created volumes, a method with previous proven high precision and accuracy. CT images of ten randomly selected patients were used. The DICOM images were converted to STL files. Three dimensional images of left hemi pelvis were reversed and merged with the right side. The posterior aspect of the pelvises was considered static and the anterior aspect as moving. Differences in translation and rotation were measured. There were no statistically significant differences between right and left hemi pelvis. The 95% confidence interval (CI) for all mean angular differences between right hemi pelvis and mirrored left hemi pelvis were − 2° to 1.5°. The 95% CI for all mean translational differences between these two objects were − 2.3 to 2.9 mm. Differences between the right hemi pelvis and the mirrored images of the left hemi pelvis for any patient greater than 3 mm or 2 degrees could be excluded with a 95% confidence. The left and right hemi pelvis of healthy adults are symmetrical enough. The pre-operative planning based on a healthy contra lateral side seems reasonable.

## Introduction

Anatomical reconstruction of pelvic fractures has been shown to affect functional outcome^1^. Using the contra lateral side of the extremities to create a template for the ipsilateral reconstruction is common practice in orthopedic surgery^2^.

Conventional radiographs are the most commonly used modality to define the symmetry of the pelvis following reconstruction, however, this technique has been criticized for a lack of precision and reproducibility^3,4^. Rotational deformity has been shown to be important in assessing pelvic symmetry but is difficult to measure^5,6^.

There is a controversy in the literature regarding whether hemi pelvises are symmetrical enough to use the contralateral (normal/uninjured) side as template to plan surgery and furthermore to 3D print implant devices. This controversy might be related to the poor reliability when using conventional radiographs or manual measurement of CT-images^7–12^.

Following introduction and implementation of 3D imaging and image fusion, a new promising method has been introduced facilitating measurement of translation and rotation of 3D images^2,13^.

This new technique offers mirrored 3D images of the contralateral pelvis alongside merging of the 3D reconstructed surfaces, thus facilitating calculation of rotational and translational differences. This technique has been shown precise and accurate in previous studies^2,13^.

We aimed to assess whether hemi pelvises are symmetrical in terms of translation and rotation using 3D reconstruction, point to point mirroring and merging of the 3D created volumes.

## Material and methods

Following NHS Health Research Authority guidelines and Local Institutional Approval at Imperial College Healthcare this study was planned and conducted. Informed consent was obtained from all subjects. To obtain patient imaging we used our institutions Picture Archiving and Communication System (PACS) office. Analysis was undertaken of all CT scans performed for major trauma patients between January to December 2018, identifying a series of 10 patients without evidence of pelvic injury. We have previously used this same study group to study femoral symmetry^2^.

A 256-slice Philips Brilliance contrast-enhanced CT scan was performed (KONINKLIJKE PHILIPS N.V., Amsterdam, Netherlands). The Gantry was AirGlide, Aperture 700 mm, Focus-isocenter distance was 570 mm and Focus-detector distance was set at 1040 mm. The rotation time was 0.27 s and Collimation was 2 × 128 × 0.625 mm. A Field of View (FOV) of 200–500 mm and matrix of 512 was used. The filter used was the iDose4 Premium Package. The tube current was set to 89–134 mAs and a dose of 520–920 mGy*cm. The average tube voltage used was 100 Kv.

**Figure 1:**
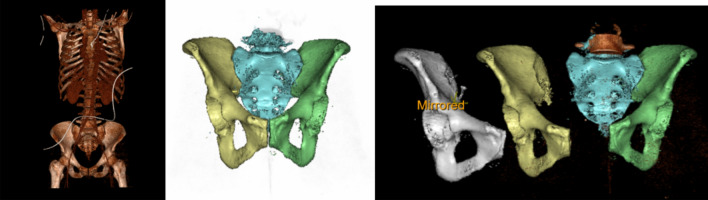
3D CT module showing the steps in the reconstruction.

All images were downloaded as Digital Imaging and Communications in Medicine (DICOM) files. Files were anonymized, coded and transferred to a research server. Stereolithographic (STL) files were created in 3D using a 3D Trauma package (SECTRA WORKSTATIONS IDS7, Version 22.1.0.1891, 2020 SECTRA AB, Linköping, Sweden, https://localhost:81/ids7/) (Fig. 1). Right and left side of the pelvises were then segmented. The left hemi pelvis was mirrored using available applications in the 3D Trauma package^2^ (Fig. 1).

**Figure 2:**
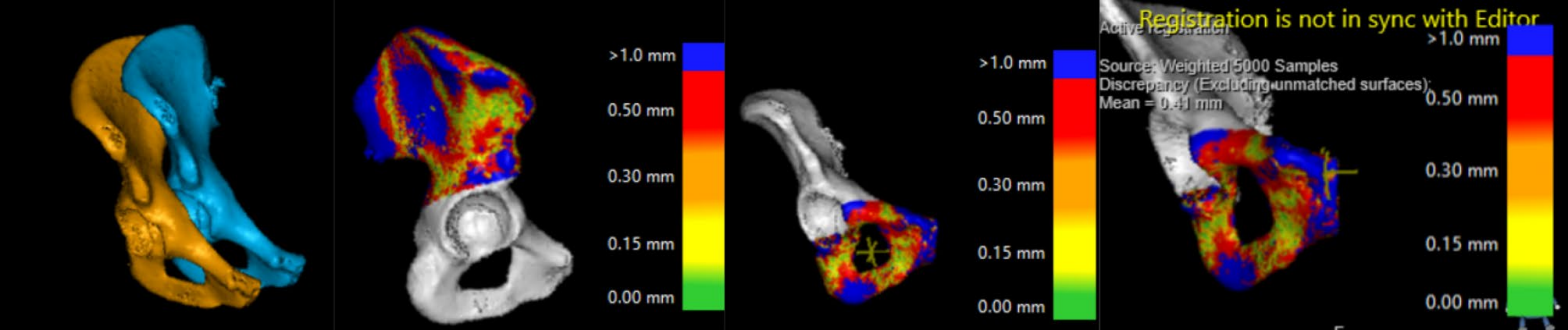
CTMA module shows steps in the volume merging. The picture to the right shows marking of a point in the anterior superior aspect of the symphysis to measure the translational differences.

Images of the right hemi pelvis and mirrored images of left hemi pelvis were saved (Fig. 2).

The software in 3D Trauma package uses two parameters to find the optimal segmentation; first the user differentiates the femur from the pelvis by clicking on their respective surfaces in the software. Secondly, the first commando in combination with the Hounsfield Unit values (HU-values) is used to find where one bone ends and the other starts (i.e. optimal segmentation). Analyses of images were done using CT Motion Analysis (CTMA). This software can precisely find the relative movement of an object between two different CT-stacks. In both CT-stacks up to 100 000 measurement points on the surface of the object of interest spreads randomly. The random points create geometrical patterns. Thereafter, the software rotates and translates the object in the second CT-stack to get the best possible match in the first CT-stack as closely as possible (Fig. 2). This procedure is done by minimizing the distance between the two groups of points. This procedure is called “Algorithmic approach for best fit of the identical surfaces” and is described by Maguire et al^14^.

As the used surfaces are much larger than any artifact areas, impact of the artifacts is limited on the matching process. The process is done first for a reference object which is defined as stationary, with which the frame of reference is created. Thereafter, the movement of the object of interest is measured in the same way and the second part is defined as the moving part. The process has been previously described in greater detail^2,13^.

Based on our previous experience from using this software, 10 000 points with a mean distance difference between meshes of 0.5 mm or less was chosen^2,13^. No smoothing was used in the CTMA software.

Translations and particularly rotations of the 3D objects in the space are measured respect another objects and references. In our proposed technique the posterior aspect of the mirrored images of the left hemi pelvis are merged with the right hemi pelvis in order to create this stationary reference volume. Further the anterior aspect of the mirrored images of the left hemi pelvis is merged with the right hemi pelvis’s counterpart and registered as a moving object. The posterior part of the right and left hemi pelvises, excluding acetabular vault and including the posterior superior iliac spine, posterior inferior iliac spine and parts of the pelvic wing of each STL created 3D volume were merged with the mirrored contralateral side (Fig. 2). These merged images were saved as static, or nonmoving parts, and were used as reference volumes. Furthermore, the anterior part of the hemi pelvises, excluding acetabulum but including superior/inferior rami were merged.

Translational and rotational changes in 3 different Euler axes (X, Y and Z) was calculated in the CTMA package^15,16^. As per DICOM standard these axes and the rotations were defined; axis X from the patient’s left to the right, axis Y from the patient’s front to back and axis Z from the patient’s feet to head. A clockwise rotation was defined as positive alongside an axis. Translational changes were reported for the entire volume of an object based on Centre of Mass (COM) or any user defined point. This COM was similar but not identical to the mathematical center of the geometric volume on which the 10 000 points were spread out. As user defined points in our study we used one point in the Anterior Superior Symphysis (ASS) and another point in the Anterior Inferior Symphysis (AIS). Rotation was reported for the entire geometrical volume.

### Statistics

Accuracy was analyzed as per Root Mean Square Error (RMSE) with mean, median and 95% confidence interval (CI) of the mean^17^. Shapiro–Wilk and Kolmogorov–Smirnov tests were used to test the distribution of normality. Statistical analysis was performed using IBM SPSS Statistics version 25 for Windows. A p-value < 0.05 was considered statistically significant.

## Results

The mean age of the study population was 54 ± 20 years. Six of the study population were males and the other four were females. There were eight cases of white British origin, one black African and one from the middle east. Differences in rotation and translation in X, Y and Z-axes of COMs are presented in Table 1. Table 2 is presenting Mean, Median and 95% CI of the measurements according to RMSE. All variables were normally distributed. Table 3 is showing normality analysis of the variables.

**Figure 3:**
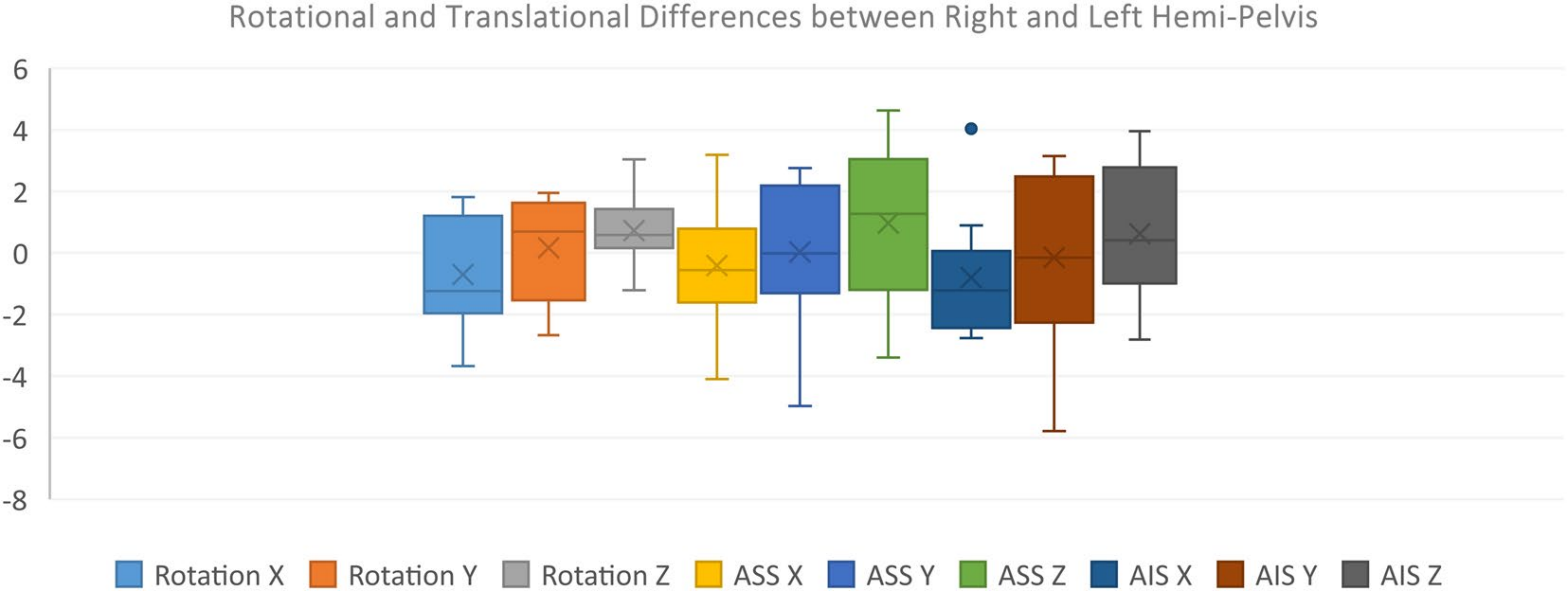
Interquartile range (IQR) of translational and rotational differences between right and left hemi pelvis) in X, Y and Z-axis for all subjects (ASS: Anterior Superior Symphysis, AIS: Anterior Inferior Symphysis).

**Figure 4:**
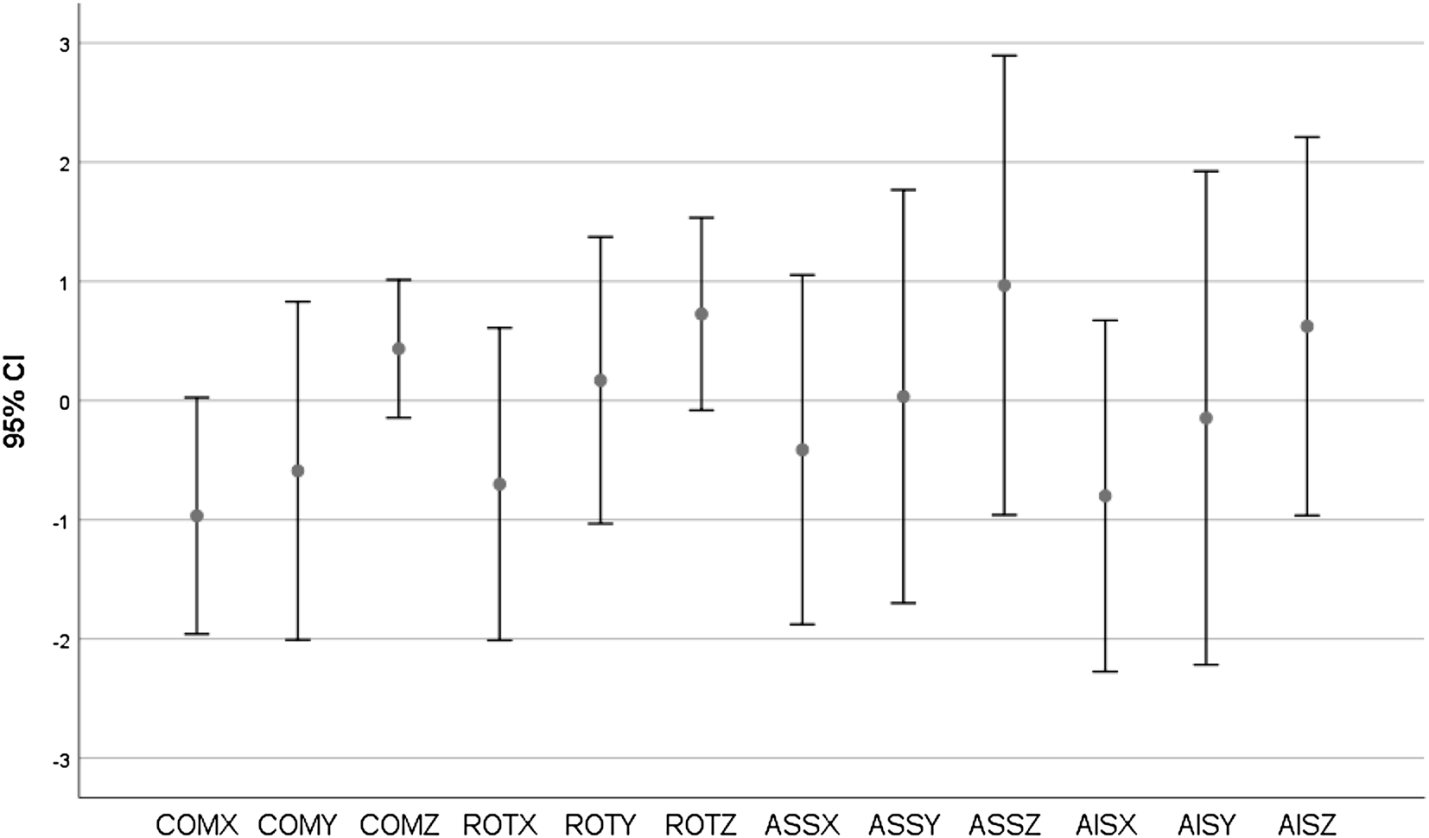
95% Confidence Interval (CI) of mean between right and left hemi pelvis in X, Y and Z-axis for all subjects (COM: Centre of Mass, ROT: Rotation, ASS: Anterior Superior Symphysis, AIS: Anterior Inferior Symphysis).

Figures 3 and 4 illustrates the normal distribution of differences alongside the coordinates showing 95% CI of the mean differences and Interquartile Range (IQR) of the differences covering 0.

The 95% for all mean angular differences between right hemi pelvis and mirrored left hemi pelvis were − 2.011 to 1.534. The 95% for all mean translational differences between these two objects were − 2.273 to 2.893. Differences between the right hemi pelvis and the mirrored images of the left hemi pelvis for any patient greater than 3 mm or 2 degrees could be excluded with a 95% confidence.

## Discussion

The main finding of our study was that the mirrored images of the left hemi pelvis were highly symmetrical with the right hemi pelvis. Differences between the right hemi pelvis and the mirrored images of the left hemi pelvis for any patient greater than 3 mm or 2 degrees could be excluded with a 95% confidence. These small differences seem to have no clinical implications for pelvic surgeons planning their surgery or templating their implants^1,18^.

Osterhoff et al. recently published an article using mirror images of contralateral pelvis and 3D templates of peri-acetabular plates in order to test symmetricity of left versus right acetabulum^19^. The authors found no statistically significant differences between right and left acetabulum when measuring the distances between a mirrored pre-contoured acetabular plate to the acetabular bone^19^. While the authors offer a new technique to define symmetricity of contralateral acetabulum based on distances, no direct measurement technique has been introduced to measure the rotational differences between the two volumes.

Badii et al., utilising CT scans alongside manual measurement found a range of asymmetry; − 11 to 7 mm difference between right and left hemi pelvis. The authors described a manual technique using the distance between the iliac crest to the acetabulum bilaterally^12^. However, manual calculations are highly subject to bias because of intra- and inter observer reliability issues. Finding reference points and reproduction of the different measurement techniques is difficult^4^. Measuring rotational differences between two hemi-pelvises has been a recurrent problematic issue in previous studies^4,6^.

In an equivalent fashion using reverse engineering Ead et al. have been successful showing that first hemi pelvises are symmetrical and further that the contra lateral hemi pelvis can be used as a surrogate for reconstruction of the injured side^20,21^. However, the authors use engineering techniques not easily available to the clinicians.

We have been able to introduce a new technique using fusion of the volume using two software packages (3D trauma and CTMA). Application of CTMA in a pelvic fracture model in a previous study showed a precision of ± 0.2 mm for translation and ± 0.2°for rotation^13^.

In this study, we used translation of two points in the symphysis pubis with respect to the merged images in the posterior aspect of the pelvises. Osterhoff et al. used the mid portion of the pelvises where the acetabulum is located. If we had merged the images posteriorly and included areas very close to the acetabulum, we would have been able to report a narrower confidence interval of the mean differences similar to that presented by the Ostehoff et al. (± 0.2 mm). However, we decided to use the pubic symphysis in our study as it was aimed to investigate the entire pelvis rather than acetabulum only^22^.

In the era of three-dimensional planning and 3D printing of pre-contoured implants, knowledge regarding symmetricity of the hemi pelvises using a highly precise technique is useful. Additionally, the technique can be used for the reconstructed pelvis to check the quality of post-operative reconstruction^8–10,23,24^.

Our study had some clear limitations. One limitation is its small sample size. The reason why we accepted this sample size was that sensitivity of the software used to detect small translations and rotations compared to what is important for the clinicians. For example, if the accuracy of the software was even ± 1 mm to detect translation and our confidence band was ± 3 mm, then a sample size as small as 6 would be enough. As we were not sure which effect size we were looking for we kept the characteristic of our study as observational. However, as our normality tests showed that our variables are coming from a normal population, increasing the sample size would probably have only contributed to a narrower confidence band. Another explanation why we did not choose a larger sample size is that the procedure at the moment is time consuming. At the moment the procedure consisting of downloading The DICOM images from the hospital’s PACS system and uploading them to the research system and optimal merging of the volumes takes roughly 3 days for each sample. Future technical development will hopefully facilitate utilization of larger sample size.

## Conclusion

Hemi pelvises of healthy adults appear to show enough symmetry to be used for pre-contouring of implants and planning of pelvic fracture surgery.

**Table 1:** Differences in rotation (degrees) and translation (mm) for each subject in X, Y and Z-axes.

Subject no	COMX (mm)	COMY (mm)	COMZ (mm)	TADX (Deg)	TADY (Deg)	TADZ (Deg)	SASX (mm)	SASY (mm)	SASZ (mm)	IASX (mm)	IASY (mm)	IASZ (mm)
1	− .761	− .435	.438	− 1.903	.187	.703	− .127	− .688	2.233	− .589	− 1.184	1.119
2	− 1.273	− .523	.405	− 2.120	.353	.463	− .835	− .629	2.520	− 1.070	− 1.083	1.790
3	− .364	1.500	1.396	1.375	1.036	.889	.359	2.700	− 1.078	− .214	3.133	− .692
4	− 1.820	.644	− .004	1.820	1.589	.282	− 2.111	1.638	− 3.395	− 2.758	2.269	− 2.814
5	− 1.427	− 1.717	− .532	− 1.722	1.052	.369	− 1.443	− 2.024	.310	− 1.837	− 2.378	− .548
6	− 2.443	− 4.453	.883	− 1.765	− 2.269	− 1.209	− 4.096	− 4.972	4.344	− 2.767	− 5.784	3.956
7	2.624	.635	.629	.589	− 2.670	− .196	3.187	.604	2.618	4.039	.775	2.853
8	− 1.638	.785	1.856	1.148	1.760	1.523	− .863	2.758	− .967	− 2.332	3.151	− .295
9	− 1.546	.992	− .761	− .758	1.952	1.395	− .280	2.020	− 1.547	− 1.361	1.857	− 1.891
10	− 1.014	− 3.315	.037	− 3.673	− 1.292	3.045	2.083	− 1.063	4.630	.893	− 2.221	2.752

Deg, degrees; COM, Centre of Mass; TAD, Total Angular Difference; ASS, Anterior Superior Symphysis; AIS, Anterior Inferior Symphysis.

**Table 2:** Median, mean and 95% CI of mean differences in rotation (degrees) and translation (mm) in X, Y and Z-axis for all subjects.

	Median	Mean	95% CI of mean
COMX (mm)	− 1.386	− 0.966	− 1.958 to 0.256
COMY (mm)	0.100	− .588	− 2.008 to 0.830
COMZ (mm)	0.421	0.434	− 0.144 to 1.013
TADX (Deg)	− 1.240	− 0.701	− 2.011 to 0.609
TADY (Deg)	− 0.694	− 0.169	− 1.032 to 1.372
TADZ (Deg)	0.583	0.726	− 0.081 to 1.534
SASX (mm)	− .557	− .412	− 1.878 to 1.052
SASY (mm)	− 0.012	0.034	− 1.698 to 1.767
SASZ (mm)	1.271	0.966	− 0.959 to 2.893
IASX (mm)	− 1.215	− 0.799	− 2.273 to 0.673
IASY (mm)	− 0.154	− 0.146	− 2.216 to 1.923
IASZ (mm)	0.412	0.623	− 0.964 to 2.210

CI, Confidence Interval; Deg, degrees; COM, Centre of Mass; TAD, Total Angular Difference; ASS, Anterior Superior Symphysis; AIS, Anterior Inferior Symphysis.

**Table 3:** Illustrates test of normality.

Tests of normality						
	Kolmogorov-Smirnov^a^			Shapiro–Wilk		
	Statistic	df	Sig	Statistic	df	Sig
COMX	.241	10	.103	.768	10	.006
COMY	.231	10	.138	.871	10	.102
COMZ	.105	10	.200*	.976	10	.943
ROTX	.211	10	.200*	.924	10	.388
ROTY	.204	10	.200*	.879	10	.127
ROTZ	.147	10	.200*	.959	10	.779
SASX	.153	10	.200*	.971	10	.902
SASY	.146	10	.200*	.923	10	.387
SASZ	.181	10	.200*	.942	10	.572
SAIX	.188	10	.200*	.858	10	.073
SAIY	.156	10	.200*	.925	10	.403
SAIZ	.160	10	.200*	.962	10	.804

^a^Lilliefors Significance Correction.

*This is a lower bound of the true significance.
